# Beyond Geography: Social Quality Environments and Health

**DOI:** 10.1007/s11205-023-03073-1

**Published:** 2023-02-03

**Authors:** Yang Li, Dario Spini, Dimitrios Lampropoulos

**Affiliations:** 1 Department of Gerontology, University of Southampton, University Road, Southampton, SO17 1BJ UK; 2 Swiss National Centre of Competence in Research LIVES, Lausanne, Switzerland; 3 Institute of Social Sciences, University of Lausanne, Lausanne, Switzerland

**Keywords:** Quality of life, Social determinants, Social well-being, Ecology, Multilevel intervention, Participatory action research

## Abstract

The concept of social quality has garnered increasing attention as a composite indicator of the well-being of societies as well as individuals embedded within them. Prior research suggests four domains of social quality: socio-economic security, social cohesion, social inclusion, and social empowerment, based on the assumption that these domains influence health and well-being. In this paper, we investigate whether and to what extent social quality environments defined with reference to the cross-cutting social quality domains reliably predict various types of health, using data collected in a municipality in Switzerland as part of a participatory action research project. We found that social inclusion had the highest predictive power for mental health and functional health, while economic security had the highest predictive power for physical capacity and overall self-rated health. Results indicate interaction among various domains of social quality for a subset of health measures. Findings suggest that environments defined as combinations of social quality domains effectively distinguish between population segments with varying levels of health. Social quality represents a promising avenue for policy and intervention development, particularly from the social determinants of health perspective, as it jointly captures the multiple domains of social well-being relevant to population health.

**Supplementary Information:**

The online version contains supplementary material available at 10.1007/s11205-023-03073-1.

## Introduction

How do we measure collective social well-being that is fundamental to health and quality of life for members of the society? The concept of social quality emerged in the 1990s against the backdrop of an overwhelming emphasis on economic growth, which was thought to improve standard of living across many societies (Beck et al., 1998). While economic growth focused on increasing material accumulation, it neglected human health, social progress, communities, institutions, and the environment, including everything “except that which makes life worthwhile” (Di Tella & MacCulloch, 2008; Mankiew, 1999). By contrast, social quality refers to the multidimensional assessment of social well-being relevant to individual health and quality of life (van der Maesen & Walker, 2005), and is based on the idea that individuals live within communities and interact with the social environment, and is thus indicative of the “quality of society” (Abbott & Wallace, 2012; Holman & Walker, 2018).

**Fig. 1:**
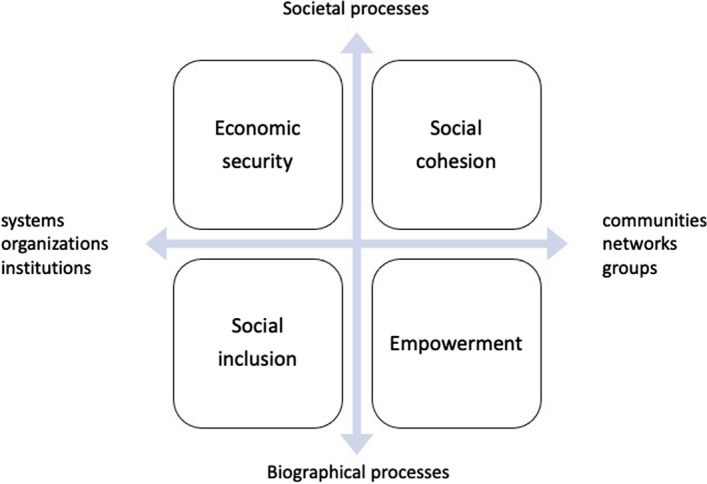
The Four Domains of Social Quality. Source: Abbott and Wallace (2012); van der Maesen and Walker (2005)

The social quality model focuses on the interactions between people’s self-realization as social beings and the formation of collective identities in the social environment (van der Maesen & Walker, 2005). Accordingly, social quality consists of four domains that are seen as necessary for social quality to develop: socio-economic security, social cohesion, social inclusion, and social empowerment, each of which represents a different dimension of the interaction between people and society (van der Maesen & Walker, 2005). As shown in Fig. 1, the four quadrants of social quality represent the relationship between societal and biographical processes (vertical axis) as well as the relationship between systems, institutions and communities (horizontal axis) (Abbott & Wallace, 2012; van der Maesen & Walker, 2005). In essence, social quality deals with the interdependencies between the social environment and the individuals who reside and carry out daily activities within such environment, capturing the ecological connections between multi-level contexts that shape people’s lives and health across time and space (Abbott & Wallace, 2012; Holman & Walker, 2018).

### Social Quality and Population Health

Recent literature suggests that the social quality model may offer theoretical enrichment for research on the social determinants of health (Holman & Walker, 2018; Ward et al., 2011), given its emphasis on the social nature of human well-being and the social production of illness. The social quality model is uniquely suited to this role for three reasons.

First, social quality is a multidimensional concept, potentially serving as a unifying model that brings together various concepts in relations to health, such as social capital (Ehsan et al., 2019), economic resources (Li & Mutchler, 2019, 2022), and social inclusion (Hartung et al., 2015), which are often independently studied within the framework of the social determinants of health (Ward et al., 2011).

Second, the social quality concept is grounded in sociological theories on the social embeddedness of human development, formally defined as “the extent to which people are able to participate in the social, economic life and development of their communities under conditions which enhance their well-being and individual potential" (Beck et al., 1998, p. 4), assuaging criticisms that research on the social determinants of health from a social epidemiological perspective often lacks a theoretical foundation (Galea & Link, 2013).

Third, the social quality model relies largely on existing indicators that are already incorporated in large datasets in many countries and that can be readily used to assess multi-domain social quality in a given a society. Abbott and Wallace (2012) comprehensively investigated social quality in more than 20 countries in Europe, using data from the European Quality of Life Surveys, covering all domains of social quality, and suggested that it was possible to operationalize the social quality model using comparative surveys across countries.

Indeed, a growing body of research from Asia (Abbott et al., 2010; Lin, 2014; Yuan & Golpelwar, 2013), Europe (Abbott & Wallace, 2012; Holman & Walker, 2018), and Oceania (Ward et al., 2011) documents the association between social quality and well-being. Holman and Walker (2018) found that various domains of social quality were associated with self-rated health in neighborhoods in Britain. Abbott et al. (2010) found that economic security had the highest predictive power for life satisfaction, followed by social cohesion and social empowerment, based on a sample in Central Asia and the Caucus. Both Lin (2014) and Yuan and Golpelwar (2013) found associations between social quality and subjective well-being in Chinese cities, noting that the various domains of social quality had differing predictive power for subjective well-being, largely concurring with Abbott et al. (2010; 2012).

### Social Structure and Geographic Location

Prior research on social quality and well-being has been dependent on geography, almost exclusively investigating social quality within geographical units: neighborhoods, districts, cities, states, and countries. This is understandable, given that social quality is designed to capture the interactions between social beings and the collective social environment and institutions, where the ‘collectiveness’ of society is most readily reflected in geographically defined areas that form natural units for observations and analyses.

However, as Holman and Walker (2018) concluded, “ultimately, individual-level experience of social quality is generally more important for individual-level health than neighbourhood-level social quality” (p. 260). The authors continued: “the fact that the neighbourhood-level coefficients were not themselves significant suggests that higher levels of neighbourhood social quality provides an extra health benefit *only to those individuals who themselves experience higher social quality*” (p. 260). This was echoed earlier by Abbott and Wallace (2012) in their study of social quality in 27 countries in Europe: “agency and the ability to build capabilities is dependent on social and geographical location as well as individual perceptions of the opportunities available to them which are in turn influenced by their position in the societal opportunity structures” (p. 155).

These observations highlight the need for expanded thinking on social quality, that is, a conceptualization based not necessarily on geographic locations, but on locations in the societal opportunity structures, given that individuals in the same geographic locations may have entirely different experiences and perceptions of social quality, largely dependent on their social location and agency. Equally, individuals in separate geographic locations may share common perceptions of social quality, given that they occupy similar locations in the social opportunity hierarchy, where social opportunities structures emphasize distributional attributes in the society (Fraser & Honneth, 2003). Research shows, for example, that perceptions of social inclusion differed considerably across population segments residing in the same municipality but occupying different social identity intersections that cut across nationality, age, gender, and educational attainment (Li & Spini, 2022). This suggests that social clusters—without reference to geographic location—may capture common exposures in the social environment in relation to health that would not be explained by spatially defined units such as neighborhoods.

### The Meso-Context of Social Quality and Health

**Fig. 2:**
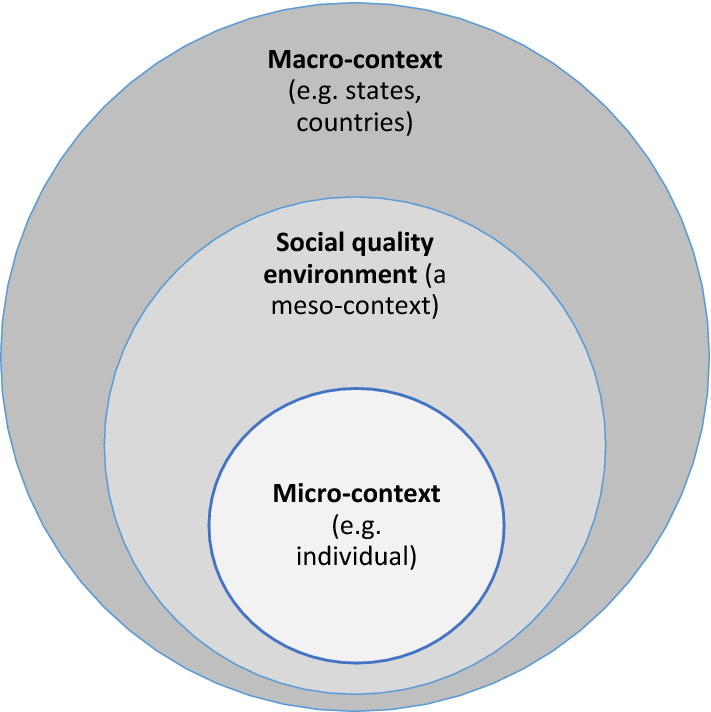
The meso-context of social quality

The concept of social clusters in relation to social quality can be explained through the socio-ecological framework for human development (Bronfenbrenner, 1979), and in particular the meso-context. Within the ecology framework, the meso-context (Fig. 2) is located between the macro-context (e.g. countries, regimes) and the micro-context (e.g. individuals, households) (Greenfield et al., 2019), representing organizations or groups sharing similar experiences or networks in relation to human development and well-being (Bronfenbrenner, 1979; Vacchiano & Spini, 2021), with or without attachment to geographic location. In the context of social quality, individuals within the same meso-context share common experiences and exposures in the social environment that condition their perceptions and experiences of social quality. Thus, the social quality environment is seen as a specific form of meso-context on the continuum of ecological contexts between the micro and macro. Based on this conceptualization, therefore, the social identity intersections in relation to social inclusion identified by Li and Spini (2022) can be seen as meso-contexts that effectively distinguish between population segments occupying different meso-contexts or social clusters, and therefore have distinct perceptions and experiences of social inclusion, despite residing in the very same municipality, and possibly in the same apartment building.

An additional advantage of the meso-context conceptualization of social quality—independent of geography—is that it allows for an investigation into the *multiplicative* nature of the social quality dimensions beyond the current emphasis on their independent and *additive* contributions to health and well-being. This is important given prior research suggesting that some of the social quality domains can *interact* and reinforce one another in real life, indicating that their multiplicative effects are potentially greater than the combination of their additive effects. For example, economic insecurity and social exclusion, two domains of social quality, are shown to be mutually enhancing, whereby poorer individuals are more likely to face social exclusion, highlighting the “spillover” cross-effects (Devicienti & Poggi, 2011) of economic security and social inclusion. Thus, consideration of the potential interactions between various social quality domains would add to our understanding of the complex social processes underpinning this important multi-domain concept.

### The Present Study

Framed within the social quality model, and drawing insights from the ecology framework regarding the meso-context, this study investigates the predictive power of meso- contexts of social quality for multiple measures of health in a local municipality with a diverse resident population in western Switzerland. The present study goes beyond the conventional approach by situating individuals within their relevant “social quality environments” and quantitatively examining the role of multidimensional social quality in shaping health and well-being. Instead of viewing socio-economic security, social cohesion, social inclusion, and social empowerment as four “quadrants” of social quality (Fig. 1), we conceptualize these four dimensions as overlapping meso-contexts representing the social interactions and processes that condition the experiences of social quality and health.

Specifically, we examine the predictive power of meso-contexts of social quality for various health outcomes based on the social locations shared by people in specific social quality environments defined with reference to the cross-cutting domains of social quality. This research addresses two focal questions: (1) Whether and to what extent are inequalities in health outcomes explained by social quality environments that cut across socio-economic security, social cohesion, social inclusion, and social empowerment? (2) How much relative predictive power does each of the aforementioned social quality dimensions have, relative to an analysis that does not account for any intersecting social quality dimension? The study identifies not only the social quality dimensions with the highest predictive power for specific health measures, but also the specific social quality environments that are most health-enhancing, offering evidence for synergistic interventions to promote health and well-being.

## Methods

### Data

Data were collected as part of the Participatory Action Research (PAR) project “Cause Commune” (i.e. common cause, or, chat in the community) from community-dwelling adults aged 18 or older in 2019 (Lampropoulos et al., 2022; Plattet & Spini, 2021). PAR is an approach aimed at engaging and promoting the competences of local residents to bring about changes in the community (Kidd & Kral, 2005; Minkler & Wallerstein, 2008). Within the context of PAR, the Cause Commune project was developed to better understand social problems and to identify intervention pathways in Chavannes-près-Renens, a municipality in the Swiss canton of Vaud, bordering Lake Geneva to the south and France to the west. Chavannes-près-Renens has a diverse population: 52% of its 8060 inhabitants (December, 2019) are of non-Swiss origins, representing nearly 100 nationalities spanning Africa, Asia, North and South Americas, the Middle East, and other parts of Europe (Plattet & Spini, 2021). To promote social integration and well-being among local residents, increasing attention is paid to better understand disparities in well-being among local residents (Plattet & Spini, 2021), and to identify intervention pathways and inform policy formulation. The Cantonal Commission on Ethics in Human Research (CER-VD) is a cantonal administrative body in Switzerland established by the Law on Human Research to ensure the protection of research subjects and to assess the compliance of human research projects with ethical, legal and scientific requirements. The CER-VD concluded that the current project did not fall within the scope of human research.

The Cause Commune project involved the entire adult resident population of Chavannes-près-Renens. With support from the municipality administration, all 6220 adults who were eligible for the survey were contacted, and 1492 individuals participated by responding to the questionnaire, yielding a participation rate of 24% (Plattet & Spini, 2021). Both paper and digital questionnaires were given to the respondents, who can respond either digitally online or by return post if using the paper version (Spini et al., 2021). The questionnaire was available in the eight most-spoken languages in the municipality—French, English, Italian, Spanish, Portuguese, Turkish, Serbian and Albanian—according to the demographic characteristics of the municipality (Spini et al., 2021). In addition, a telephone line was set up to allow participants to directly request a paper version of the questionnaire in the language of their choice (Spini et al., 2021). Among the 1492 responses, 91 questionnaires were withdrawn due to incomplete responses or duplicate participations revealed by identical personal codes. Thus, the final analytic sample consisted of 1401 adult residents aged 18 or older in Chavannes-près-Renens.

## Measures

### Health Outcome

We measured five health domains using questions inspired by the 12-Item Short-Form Health Survey (Ware et al., 1996). A detailed questionnaire can be found in Appendix 1 (supplementary material). All measures of health were coded such that a higher number indicated better health (1 = worst to 5 = best). *General health* was measured as self-rated health status from poor to excellent, which has been widely used to assess health outcomes and health disparities (Idler & Benyamini, 1997; Jylhä, 2009). *Physical functioning* was measured using four items (α = 0.88) concerning the ability to perform household chores, walk for 10 min, climb up stairs, and play sports in the past 4 weeks (Gandek et al., 1998). *Role limitation due to physical problems* was evaluated using two items (α = 0.93) asking respondents whether they “accomplish less things than desired” and “have been limited in work and activities” due to physical health in the past 4 weeks (Gandek et al., 1998). *Role limitation due to emotional problems* was assessed using two items (α = 0.93) asking respondents whether they “accomplish less things than desired” and “have been limited in work and activities” due to emotional problems in the past 4 weeks (Gandek et al., 1998). Finally, *mental health* was measured using three questions (α = 0.92) asking respondents how often they felt “calm and peaceful”, “full of energy”, and “downhearted or discouraged” in the past 4 weeks (Rumpf et al., 2001).

### Social Quality

We measured all four domains of social quality using survey questions already established in prior literature (van der Maesen & Walker, 2005). A detailed questionnaire containing specific question wording and response options for all measures of social quality can be found in Appendix 2 (supplementary material). All measures were coded such that a higher number indicates greater social quality. *Economic security* was measured using four questions (α = 0.74) on material scarcity, income satisfaction and comparison, based on questions developed in MOSAiCH (Measurement and Observation of Social Attitudes in Switzerland), a survey of the Swiss population’s values and attitudes on social issues (MOSAiCH, 2017). *Social support* was assessed using three questions (α = 0.73) asking respondents whether they can “obtain advice in neighborhood”, “borrow things from neighbors”, and “talk regularly to neighbors” (University of Essex, n.d.). *Social inclusion* was evaluated using four questions (α = 0.75) regarding social and emotional loneliness (De Jong Gierveld & Van Tilburg, 2010). *Social empowerment* was assessed with five questions (α = 0.90) on respondents’ ability to improve neighborhood’s living, help with its organization, intervene in local decision-making, make a specific request to the municipality, and whether the community can cooperate in times of difficulty (Zimmerman, 1995).

To operationalize the social quality “environments” conceptualized in this paper based on respondents’ cross-classified attributes, all measures of social quality were coded as tertiles (high, mid, low) so that the respondents were roughly evenly distributed across the tertiles for each domain of social quality. The final analytical sample consists of 1,342 individuals nested within 81 (= 3^4^) strata, after 59 cases (4%) were dropped due to missing data (Allison, 2001) on any of the domains used to construct the 81 strata because these cases cannot be nested in any stratum. The average number of observations per strata was 17.

### Control Variables

All analysis controlled for age, gender, and education. Gender was coded binary (0 = male, 1 = female). Age was categorized into three groups: 18–40, 41–64, 65 + , with attention to the differential life roles by age groups (Settersten, 2003) and the analytical benefits of distributing the sample roughly evenly between these categories. Educational attainment was classified as primary, secondary, and tertiary (Federal Statistical Office, 2020).

### Analytic Strategy

We used the multilevel analysis of individual heterogeneity and discriminatory accuracy —a set of models that partition the total variance into between-strata and within-strata—to estimate the predictive power of social quality environments for health (Merlo, 2018). This novel and innovation technique is uniquely suited to address our research questions because it allows for an ecological examination of health inequities by partitioning the total variance into micro- and meso- levels, making it possible to evaluate the interlocking dimensions of social quality (Evans et al., 2018; Li, 2022). This approach nests each individual in their relevant strata—e.g. high social inclusion *and* low economic security *and* mid empowerment *and* high social support —and estimates random effects at the stratum level. To investigate the relative predictive power of each social quality domain, we used the same technique and analyzed the proportional change in variance between a reference model and the model with an added dimension of interest (e.g. inclusion) (Persmark et al., 2019). The larger the proportional change in the random intercepts’ variance between the two models, the greater predictive power that added dimension has. The theoretical foundation, empirical strategies and advantages of this technique have been extensively documented in the literature (Evans et al., 2018; Merlo, 2018; Persmark et al., 2019). No evidence of multicollinearity was found (VIF < 2 for predictors).

## Results

**Table ****1** reports the descriptive characteristics of the study sample. Respondents reported fairly good average levels of health overall (1 = worst health to 5 = best health): self-rated health (3.9), physical functioning (4.0), role emotional (4.0), role physical (4.2), and mental health (3.6). In terms of social quality domains (1 = worst to 5 = best), the mean level of social inclusion was about 3.7, and the mean levels of social support, empowerment, and economic security were approximately 3.1. About 54% were female. Approximately 39% aged 18–40, 39% aged 41–64, and 21% aged 65 or older. In terms of educational attainment, 10% of the respondents obtained primary education, 50% secondary education, and 40% obtained tertiary education, where tertiary education includes university education as well as professional training in the Swiss context (Federal Statistical Office, 2020).

**Table 1:** Study sample characteristics

	M	SD	%
Health outcomes (1 = worst to 5 = best)			
General health	3.9	0.9	
Physical functioning	4.0	1.1	
Role emotional	4.0	1.0	
Role physical	4.2	1.1	
Mental health	3.6	0.7	
Social quality dimensions (1 = worst to 5 = best)			
Social inclusion	3.7	0.8	
Low			33.8
Mid			41.1
High			25.1
Social support	3.1	0.8	
Low			35.6
Mid			31.2
High			33.2
Empowerment	3.1	0.7	
Low			36.9
Mid			31.2
High			32.0
Economic security	3.1	0.7	
Low			34.7
Mid			32.9
High			32.4
Demographic characteristics			
Gender			
Male			46.1
Female			53.9
Age			
18–40			39.3
41–64			39.4
65 +			21.2
Education			
Primary			9.9
Secondary			50.2
Tertiary			39.8
N = 1342			

Table 2 provides results for multilevel models on social quality environments and health. Results showed that the predictive power of the social quality domains differed by measures of health. In particular, economic security had the highest predictive power for general health and “role physical” (role limitations due to physical problems) given that the inclusion of economic security reduced the random intercept’s variance by 46.3% for general health (Table 2A, Model 4) and 46.8% for role physical (Table 2D, Model 4), respectively, relative to the null model (Model 0) where the additive effects of social quality domains were not considered. By contrast, social inclusion had highest predictive power for physical functioning, role emotional (role limitations due to emotional problems), and mental health, given that adding social inclusion reduced the random intercept’s variance by 63.1% (Table 2B, Model 1), 80.8% (Table 2C, Model 1), and 71.1% (Table 2E, Model 1), respectively, relative to the null model (Model 0). When all dimensions were included in the full model (Model 5), the random intercept’s variance was reduced substantially but not completely for any of the health measures. This suggests that a portion of the between-strata variation remains unexplained by the additive effects of the social quality domains, but was captured by intersectional interaction. In particular, 11.3% of the variance in general health (Table 2A, Model 5) between social quality environments remains unexplained after accounting for the additive effects of all social quality domains. Similarly, the share of the unexplained variance for other measures of health was: 6% for physical functioning (Table 2B, Model 5), 2% for role limitation due to emotional problems (Table 2C, Model 5), 26% for role limitation due to physical problems (Table 2D, Model 5), and 8% for mental health (Table 2E, Model 5). This implies that domains of social quality have interacted to shape health for people at unique intersections of social locations who share a common set of social exposures such as norms, opportunities, and constraints relating to health. While we acknowledge that the unexplained portion of the between-environment variation is a share of an initially small total variance (ICC = 6.2% for general health, Table 2A, Model 0), it is however non-trivial and adds to our understanding of the non-additive patterning of social quality.

**Table 2:** Multilevel analysis for indicators of health within social quality environments

Social quality domains	0	1	2	3	4	5
*A. General health*						
Social inclusion		0.16 (0.08, 0.24)				0.14 (0.07, 0.20)
Social support			0.11 (0.03, 0.19)			0.08 (0.01, 0.14)
Empowerment				0.08 (-0.01, 0.16)		0.05 (-0.01, 0.12)
Economic security					0.18 (0.10, 0.26)	0.17 (0.11, 0.24)
Between-environment variance	0.050	0.032	0.041	0.048	0.027	0.006
ICC (%)	6.21	4.04	5.07	6.00	3.44	0.74
PCV (%)	Ref	− 36.53	− 19.16	− 3.99	− 46.31	− 88.68
*B. Physical functioning*						
Social inclusion		0.22 (0.14, 0.30)				0.20 (0.13, 0.28)
Social support			0.05 (− 0.04, 0.14)			0.02 (− 0.05, 0.09)
Empowerment				0.05 (− 0.05, 0.14)		0.03 (− 0.04, 0.11)
Economic security					0.19 (0.10, 0.27)	0.17 (0.10, 0.24)
Between-environment variance	0.054	0.020	0.052	0.054	0.032	0.003
ICC (%)	5.29	2.02	5.07	5.21	3.15	0.33
PCV (%)	Ref	− 63.06	− 4.31	− 1.10	− 41.73	-94.04
*C. Role limitation due to emotional problems*						
Social inclusion		0.31 (0.23, 0.38)				0.28 (0.22, 0.35)
Social support			0.11 (0.01, 0.20)			0.07 (0.00, 0.13)
Empowerment				0.07 (− 0.02, 0.17)		0.05 (− 0.01, 0.12)
Economic security					0.11 (0.02, 0.21)	0.11 (0.04, 0.17)
Between-environment variance	0.072	0.014	0.061	0.069	0.062	0.001
ICC (%)	7.69	1.57	6.67	7.43	6.73	0.02
PCV (%)	Ref	− 80.80	− 14.08	− 3.66	− 13.31	− 98.04
*D. Role limitation due to physical problems*						
Social inclusion		0.17 (0.08, 0.25)				0.15 (0.07, 0.23)
Social support			0.03 (− 0.06, 0.12)			0.00 (− 0.07, 0.08)
Empowerment				0.01 (− 0.08, 0.11)		0.01 (− 0.07, 0.09)
Economic security					0.18 (0.10, 0.26)	0.16 (0.09, 0.24)
Between-environment variance	0.045	0.025	0.044	0.044	0.024	0.012
ICC (%)	4.19	2.40	4.12	4.11	2.27	1.13
PCV (%)	Ref	− 43.67	− 1.43	− 1.65	− 46.80	− 73.71
*E. Mental Health*						
Social inclusion		0.29 (0.23, 0.35)				0.27 (0.22, 0.32)
Social support			0.08 (0.00, 0.16)			0.06 (0.01, 0.11)
Empowerment				0.11 (0.03, 0.19)		0.10 (0.05, 0.15)
Economic security					0.09 (0.01, 0.18)	0.09 (0.04, 0.14)
Between-environment variance	0.070	0.020	0.064	0.063	0.064	0.006
ICC (%)	15.07	4.87	14.04	13.83	13.94	1.42
PCV (%)	Ref	− 71.06	− 7.70	− 9.38	− 8.51	− 91.87

Estimates and 95% CIs (in parentheses) are shown

All models control for gender, age, and education

I*CC* intra-class correlation; *PCV* proportional change in variance

## Discussion

Despite a growing body of literature on social quality and health, prior research has been largely dependent on geography, attaching social quality to spatial units such as cities, states, and countries. Yet, it remains unclear whether and to what extent environments unattached to geography capture variabilities in social quality, particularly given research evidence suggesting that perceptions of social quality depend considerably on the individual and his position in the social structure. The present study extended prior literature on social quality and health by conceptualizing social quality as a meso-context, independent of geographic location, to estimate the predictive power of “social quality environments” on indicators of health in a municipality in Switzerland. Framed within the social quality model (van der Maesen & Walker, 2005), and drawing insights from ecological theories (Bronfenbrenner, 1979), this study investigated whether and to what extent social quality environments defined with reference to the cross-cutting domains of social quality reliably predict physical, mental, and functional health, utilizing data collected as part of a participatory action research project.

The study found that social quality environments meaningfully distinguished between population segments with varying levels of physical, functional, and mental health, controlling for common individual-level characteristics such as gender, age, and educational attainment. Unlike prior research, social quality environments in the present study were constructed solely based on individual perceptions of social quality, without regard to a person’s geographic location. Given that the social quality environments organized the resident population into groups with varying levels of health, policies and interventions to promote health in the community may wish to consider social quality through the lens of the “receiver”—that is, the individual who experiences social quality—to determine which social quality domains are less health-enhancing and would thus require intervention.

The study also found that the various domains of social quality had differing predictive power for health, largely concurring with prior research (Abbott et al., 2010, 2012; Lin, 2014; Yuan & Golpelwar, 2013). In particular, economic security is important for general health as well as physical health, while social inclusion is paramount for mental and functional health, including role limitations due to emotional problems. While the present study does not investigate the mechanisms that underlie these associations, prior research suggests that the link between economic security and health may be underpinned by material mechanisms where economic security impacts health through access to health-enhancing facilities and resources (Li & Mutchler, 2022), as well as psychosocial mechanisms where individuals draw comparisons based on their socioeconomic position, which in turn exert influence on health (Dunn et al., 2006). The association between social inclusion and mental health and functional health is potentially underpinned by behavioral and psychosocial mechanisms relating to reduced social participation and perceived stress from social exclusion and their impact on health (Segrin & Passalacqua, 2010), although further research is needed to elucidate these pathways. The study additionally showed that a portion of between-environment variance remained unexplained after accounting for the additive effects of all social quality domains and demographic controls, suggesting that the social quality domains of economic security, social inclusion, social support, and empowerment have interacted to shape health, pointing to potential spillover cross-effect.

A few limitations warrant discussion. First, no causal interpretations should be made from this cross-sectional study. Second, while the present study focused on social quality environments defined with reference to the cross-cutting social quality domains independent of geographic location, it is possible that a combination of social and spatial locations—i.e. socio-spatial units—would have even higher predictive power for health. Given the relatively small sample size in this study, it was not feasible to operationalize a socio-spatial approach where social quality attributes would be jointly defined based on social and spatial locations. We acknowledge that people with lower health or socioeconomic statuses may be less likely to respond. Lastly, given data limitations, it was not possible to explore other important health/illness domains such as diabetes, cancer, and mortality risks. Future research should examine more comprehensive measures of health to further elucidate the predictive power of social quality.

Despite these limitations, the present study, conceptually grounded in the social quality model and drawing insights from ecological theories, demonstrates the extent to which social quality environments predict health in a community context. Social quality environments, defined according to the cross-cutting domains of economic security, social support, social inclusion, and social empowerment, effectively distinguish between population segments in terms of their physical, functional, and mental health. Policies and interventions seeking to reduce health disparities may wish to consider priorities in resource distribution by taking into account the relevant importance of each social quality domain with respect to the specific type of health in question. Importantly, given the unique advantages of social quality as a sociologically oriented, multi-domain, and unifying framework that brings together various concepts in relation to the social production of health, the social quality model has potential to offer theoretical enrichment for the literature on the social determinants of health. Future research into the social quality model should explore the role of social identities (e.g. gender, age, race/ethnicity) in perceived social quality. Future research into social quality and health disparities within a community context should explore a socio-spatial approach where social locations and geographic locations are jointly considered in order to inform policies and interventions with greater precision.

## Supplementary Information

Below is the link to the electronic supplementary material.

Supplementary file1 (DOCX 26 KB)
