# Supplementary material for: Beyond Geography: Social Quality Environments and Health
Source: Soc Indic Res. 2023 Feb 3;166(2):365–79. doi: 10.1007/s11205-023-03073-1 (PMC10011288; doi:10.1007/s11205-023-03073-1)
Supplement: Supplementary file 1 — Supplementary file1 (DOCX 26 KB) [file 11205_2023_3073_MOESM1_ESM.docx]

Appendix 1. Questionnaire used to assess health

**(General health)**

In general, would you say your health is…?

| - Very good | - Good | - Medium | - Poor | - Very poor |
| --- | --- | --- | --- | --- |

**(Physical functioning)**

In the last 4 weeks, has your health limited you in the following activities:

|  | Never | Rarely | Sometimes | Often | All the time |
| --- | --- | --- | --- | --- | --- |
| - Cleaning up the house, shopping, gardening, DIY? | ☐ | ☐ | ☐ | ☐ | ☐ |
| - Walking at least 10 minutes consecutively? | ☐ | ☐ | ☐ | ☐ | ☐ |
| - Climbing up several flights of stairs? | ☐ | ☐ | ☐ | ☐ | ☐ |
| - Playing sports: running, dancing, soccer, ...? | ☐ | ☐ | ☐ | ☐ | ☐ |

**(Role limitation due to physical problems)**

In the past 4 weeks, how often have you had the following problems with your work or other regular activities as a result of your PHYSICAL HEALTH?

|  | Never | | Rarely | | Sometimes | | Often | | All the time | | |
| --- | --- | --- | --- | --- | --- | --- | --- | --- | --- | --- | --- |
| - Accomplished less than you would like. | | ☐ | | ☐ | | ☐ | | ☐ | | ☐ |  |
| - Were limited in your work or other activities. | | ☐ | | ☐ | | ☐ | | ☐ | | ☐ |  |

**(Role limitation due to emotional problems)**

In the past 4 weeks, how often were you limited in your work or other regular activities as a result of any EMOTIONAL PROBLEMS?

|  | Never | Rarely | Sometimes | | Often | All the time |
| --- | --- | --- | --- | --- | --- | --- |
| - Accomplished less than you would like. | ☐ | ☐ | ☐ | ☐ | | ☐ |
| - Didn’t do work or other activities as carefully as usual. | ☐ | ☐ | ☐ | ☐ | | ☐ |

**(Mental health)**

In the last 4 weeks, how many times have you felt:

|  | Never | Rarely | Sometimes | | | Often | All the time |
| --- | --- | --- | --- | --- | --- | --- | --- |
| - Calm and peaceful ? | ☐ | ☐ | | ☐ | ☐ | | ☐ |
| - Full of energy? | ☐ | ☐ | | ☐ | ☐ | | ☐ |
| - Downhearted or discouraged? | **☐** | **☐** | | **☐** | **☐** | | **☐** |

Ware Jr, J. E., Kosinski, M., & Keller, S. D. (1996). A 12-Item Short-Form Health Survey: construction of scales and preliminary tests of reliability and validity. *Medical Care*, *34*(3), 220-233.

Appendix 2. Questionnaire used to assess social quality

**(Economic security)**

1. Considering all the income and expenses of your household (a household is defined as all the people who regularly share the same roof and meals) over a year, would you say that currently your household....:

| ☐ manages to save a lot of money ☐ manages to save money ☐ spends what it earns  ☐ consumes its assets and reserves ☐ goes to indebtedness |
| --- |

1. Regarding your income, do you think you are

| Well below average ☐ | Below average ☐ | In the average ☐ | Above average ☐ | Far above average ☐ |
| --- | --- | --- | --- | --- |

1. To ensure your needs, how do you see your current financial situation?

| Very unsatisfactory ☐ | Unsatisfactory ☐ | Average ☐ | Satisfactory ☐ | Very satisfactory ☐ |
| --- | --- | --- | --- | --- |

1. How easy or difficult is it for you or your household to complete or "make ends meet"?

| Very difficult | Quite difficult | Neither difficult nor easy | Quite easy enough | Very easy |
| --- | --- | --- | --- | --- |
|  |  |  |  |  |

**(Social support)**

| In my neighbourhood… : | Strongly disagree | Disagree | More or less | Agree | Strongly agree |
| --- | --- | --- | --- | --- | --- |
| 1. I can get advice. | ☐ | ☐ | ☐ | ☐ | ☐ |
| 1. I can borrow things from my neighbours. | ☐ | ☐ | ☐ | ☐ | ☐ |
| 1. I regularly talk to my neighbours. | ☐ | ☐ | ☐ | ☐ | ☐ |

**(Social inclusion)**

|  | Strongly disagree | Disagree | More or less | Agree | Strongly agree |
| --- | --- | --- | --- | --- | --- |
| 1. I miss having people around me. | ☐ | ☐ | ☐ | ☐ | ☐ |
| 1. I often feel rejected. | ☐ | ☐ | ☐ | ☐ | ☐ |
| 1. There are many people I can trust completely. | ☐ | ☐ | ☐ | ☐ | ☐ |
| 1. There are enough people I feel close to. | ☐ | ☐ | ☐ | ☐ | ☐ |

**(Social empowerment)**

|  | Not at all | No | More or less | Yes | Absolutely |
| --- | --- | --- | --- | --- | --- |
| 1. Do you feel able to partner others to improve your neighbourhood’s living? | ☐ | ☐ | ☐ | ☐ | ☐ |
| 1. Would you agree to help with the organization of a group activity in your neighbourhood or town? | ☐ | ☐ | ☐ | ☐ | ☐ |
| 1. Do you think that your community is able to cooperate facing difficulties in order to improve your neighbourhood’s living? | ☐ | ☐ | ☐ | ☐ | ☐ |
| 1. Do you feel able to intervene in the decision-making process impacting the municipality (e.g. writing to the municipality, propose a project)? | ☐ | ☐ | ☐ | ☐ | ☐ |
| 1. Do you feel entitled to make a request to the municipality (e.g. restore a playground)? | ☐ | ☐ | ☐ | ☐ | ☐ |
